# PET-MRI in idiopathic inflammatory myositis: a comparative study of clinical and immunological markers with imaging findings

**DOI:** 10.1186/s42466-022-00213-9

**Published:** 2022-10-10

**Authors:** Manu Santhappan Girija, Ravindu Tiwari, Seena Vengalil, Saraswati Nashi, Veeramani Preethish-Kumar, Kiran Polavarapu, Karthik Kulanthaivelu, Arpana Arbind, Mainak Bardhan, Akshata Huddar, Gopikrishnan Unnikrishnan, Valasani Ravi Kiran, Tanushree Chawla, Bevinahalli Nandeesh, Chandana Nagaraj, Atchayaram Nalini

**Affiliations:** 1 Department of Neurology, National Institute of Mental Health And Neurosciences (NIMHANS), Hosur Road, Bengaluru, Karnataka 560029 India; 2 Children’s Hospital of Eastern Ontario Research Institute; Division of Neurology, Department of Medicine, The Ottawa Hospital, Brain and Mind Research Institute, University of Ottawa, Ottawa, ON Canada; 3 Department of Neuro Imaging and Interventional Radiology, National Institute of Mental Health And Neurosciences (NIMHANS), Bangalore, Karnataka 560029 India; 4 Department of Neuropathology, National Institute of Mental Health And Neurosciences (NIMHANS), Bangalore, 560029 India

**Keywords:** PET-MRI, Inflammatory myositis, MRI, Myositis specific antibody, Myositis associated antibody, Malignancy

## Abstract

**Background:**

We sought to determine the utility of PET-MRI in diagnosing Idiopathic Inflammatory Myositis (IIM), and look for association between FDG uptake and clinical, pathological and laboratory parameters.

**Methods:**

A retrospective, observational study was conducted on IIM patients having positive serum autoantibodies and who underwent PET-MRI (3-Tesla SIEMENS Biograph MR scanner) between 2017 and 2021. Thirty patients who underwent PET-MRI to detect systemic metastasis without muscle involvement formed the control group.

**Results:**

In the IIM cohort, female: male sex ratio was 1.73, mean age at diagnosis was 40.33 years, and the mean duration of illness was 7 months. 33.33% of patients had severe limb weakness. Mi2B (43.33%), Mi2A (43.33%), PL-7(10%), PL-12(6.67%), SRP (16.67%), Tif1gamma (3.33%), NxP2 (3.33%), Ro-52(40%), PM-Scl, U1-RNP, ANA (26.67%) were the serum autoantibodies identified. Using SUV max Ratio to quantify FDG uptake, PET-MRI showed a sensitivity of 100% with 93.3% specificity in diagnosing IIM.FDG uptake was maximum in proximal lower limb region followed by proximal upper limb. Multivariate regression analysis showed that the severity of muscle weakness, serum Mi2B antibody positivity and serum creatinine kinase levels had a significant positive correlation with FDG uptake (value of 0.005, 0.043, 0.042, respectively for whole-body FDG uptake). FDG uptake also showed good correlation with histopathological features and muscle MRI, but there was no significant association with treatment response. Three female patients in our cohort had primary malignancy involving the breast, uterus, and cervix.

**Conclusions:**

PET-MRI is a promising diagnostic modality for IIM. PET-MRI reflects the severity of muscle inflammation, showing good association with various clinical/laboratory parameters, histopathology, and muscle MRI. Parameters associated with severe muscle inflammation in PET-MRI—clinical severity of muscle weakness, Mi2B positivity, and serum creatine kinase levels—may be used as clinical/laboratory markers of disease severity in IIM. PET-MRI has the added advantage of detection of systemic malignancy.

**Supplementary Information:**

The online version contains supplementary material available at 10.1186/s42466-022-00213-9.

## Introduction

Idiopathic Inflammatory Myopathy (IIM) is classically constituted by dermatomyositis (DM), (immune mediated) necrotizing myopathy (NM), overlap syndrome with myositis (overlap myositis, OM) including anti-synthetase syndrome (ASS), polymyositis (PM), and inclusion body myositis (IBM). IIM is characterized by muscle inflammation and weakness and other organ involvement often leading to severe impairment in the quality of life [1]. OM constitutes up to 50% of IIM in various series, whereas dermatomyositis constitutes around 30%, necrotizing myositis around 10–15%, PM and IBM less than 5% each [2, 3]. With the discovery of Myositis Specific and Myositis Associated Antibodies, recently IIM is classified based on clinicoseropathological features into—DM, NM, IBM and Antisynthetase Syndrome, with questionable existence of Polymyositis as a separate entity. These subsets of IIMs have distinct immunopathogenesis, and therapeutic responses making their accurate diagnosis important [4].

Magnetic resonance imaging (MRI) of skeletal muscle can identify areas of muscle inflammation, damage, fatty replacement and fibrosis [5, 6]. [18F] fluorodeoxyglucose positron emission tomography/computed tomography ([18F] FDG-PET/CT or MRI) is a non-invasive imaging modality that combines metabolic evaluation and morphological correlation and is a standard tool for detecting malignancies. FDG is transported into cells by glucose transporters and is phosphorylated to 18F-2-FDG-6 phosphate by hexokinase enzyme which is not further metabolized. The degree of cellular FDG uptake is related to the rate of cellular metabolism and the number of glucose transporters. In active inflammatory conditions, the inflammatory cells show increased FDG uptake mediated through an increase in number as well as FDG affinity of these glucose transporters mediated through various cytokines and growth factors [7–9]. and these cells utilize glucose as an energy source only after activation during the metabolic burst [10]. Recently [18F]—FDG-PET combined diagnostic computed tomography (CT), or MRI is gaining interest in diagnosing many infectious and inflammatory conditions [10, 11]. PET imaging in IIM offers the potential advantage of malignancy screening and disease activity evaluation in a single highly sensitive test [12].

The role of FDG-PET-CT in evaluating IIM has been explored in only a few studies and none using FDG-PET-MRI, and the overall sensitivity is less compared to muscle MRI [13–17]. We sought to determine the utility of PET-MRI in diagnosing IIM and assess the correlation between FDG uptake and various clinical, pathological, and laboratory parameters. To the best of our knowledge, this is the first study in English literature to evaluate the utility of FDG-PET-MRI in IIM.

## Methodology

Institutional Ethics Committee approval was obtained for the study (NIMH/DO/IEC (BS & NS DIV)/2018-19). Thirty random patients clinically diagnosed with IIM in a tertiary Neurology specialist hospital in South India (NIMHANS, Bengaluru), having positive serum autoantibodies and who underwent PET-MRI either before or shortly after (within 10 days) pulse dose of immunosuppressant (IV Methylprednisolone), between January 2017 and January 2021, were included in the study. Clinical, laboratory and pathological data were collected retrospectively from hospital records, and imaging data were obtained from hospital PACS (Picture Archiving and Communication System). Thirty patients who underwent PET-MRI as part of the diagnostic workup for carcinoma of unknown primary or systemic metastasis without any clinical evidence of muscle involvement were taken as a control group.

IIM was diagnosed based on clinical and laboratory features. Patients who presented with typical symmetric proximal limb muscle weakness of acute to subacute onset or a relapsing course, elevated serum Creatine Kinase levels, and positive myositis antibodies constituted the disease group. Open muscle biopsy was performed in 10 patients. IIM group was categorized into 4 subgroups based on clinical features, presence of particular myositis antibodies and histopathology reports wherever available. IIM patients with either characteristic skin manifestations or Mi2b antibodies or typical histopathology findings were categorized as Dermatomyositis. Skin manifestations considered as a qualifier for DM include Gottron's papules—erythematous to violaceous papules and plaques over the extensor surfaces of the metacarpophalangeal and interphalangeal joints; heliotrope rash consisting of violaceous erythema of the upper eyelids often with associated edema and telangiectasia; erythematous patches and/or plaques in malar distribution, forehead, lateral face, and ears; confluent macular erythema over the lower anterior neck and upper anterior chest ("V" sign) and erythema over the upper back, posterior neck, and shoulders (shawl sign). Polymyositis group includes those with typical histopathology findings (muscle fiber infiltration by mononuclear inflammatory cells with absent inclusion bodies) with or without anti-SRP positivity. Finger flexor weakness, presence of inclusion bodies in muscle biopsy, and poor response to immunotherapy were used as criteria to rule out Inclusion Body Myositis. Patients with characteristic histopathological findings of muscle fiber necrosis with paucity of inflammatory infiltrates in muscle biopsy with or without anti-SRP positivity constituted IMNM group. IIM patients with associated clinical features of Systemic Sclerosis, SLE, MCTD, Rheumatoid arthritis or Sjogren’s Syndrome constituted overlap myositis group. Apart from general features of myositis, presence of perifascicular atrophy was considered diagnostic of Dermatomyositis; myofibre invasion by mononuclear inflammatory infiltrates in the absence of inclusion bodies was suggestive of Polymyositis; scattered myofibre necrosis in the absence of inflammatory infiltrates suggestive of IMNM. MRI of lower limbs was done in 13 patients to identify inflammation and also to assess the disease severity. Patients underwent PET-MRI as part of malignancy workup.

### Clinical examination

Patient data entered in pre-designed elaborate case records were scrutinized, and relevant clinical, biochemical, and electrophysiological data were collected. The severity of muscle weakness in the limbs was graded using a modified MRC scale. Grade 5 power was given score 0, grade 4 + was score 1, < 4 + but greater than grade 2 was score 2 and ≤ 2 was score 3. The scores of the limbs were added to obtain a composite clinical score that reflects the degree of muscle weakness. Patients were further subdivided into two clinical subgroups based on the total calculated score, i.e., group 1 = mild to moderate limb weakness (clinical score 0 to 8) and group 2 = severe limb weakness (clinical score 9 to 12).

### FDG PET MRI muscle

Image acquisition: All the patients underwent 18F FDG-PET-MRI on Biograph mMR (Siemens, Erlangen, Germany) after 4–6 h of fasting. Imaging was performed after 45 to 60 min of intravenous injection of 305 ± 20 MBq 18F-FDG. The images were acquired in 7 bed position (5 min/per bed) from the base of the skull to the ankle with a hands-down position to cover the maximum extent of the muscles of all 4 limbs using body coils and DIXON GRAPPA as MR attenuation sequence (MRAC). Brain PET-MRI was acquired in a single bed position (10 min) with UTE as MRAC sequence after whole body PET-MRI acquisition. All images are acquired in 3D static mode and reconstructed in axial, coronal, and sagittal planes using the following reconstruction parameters OSEM 4D, 3 iterations 21 subsets, 172 matrix, FWHM 2 mm with Gaussian filter.

Myositis was analysed by a nuclear medicine expert (CN) blinded for disease group, using PET-MRI in 10 regions which include the below muscles: sternocleidomastoids, cervical spinal, proximal and distal upper limbs, thoracic, abdominal, paraspinal, gluteal, proximal lower limb and calf muscles. FDG uptake in each of these regions was quantified using the parameter of Standardized Uptake Value (SUV). SUV is a semi-quantitative measure and is defined as the ratio of the concentration of radiopharmaceutical tracer (FDG) per unit volume of a region of interest (ROI) to concentration in the body if uniformly distributed (determined by a standard body phantom). ROI is placed in the area of maximum uptake identified through visual assessment in each muscle region. SUV is calculated as both maximum (SUV max) and mean (SUV mean) value in these ROIs. We derived two other parameters based on SUV for enabling inter-patient comparison: 1). SUV max Ratio–ratio between SUV max in ROI and SUV mean of reference region (Aorta) and 2). SUV mean Ratio–ratio between SUV mean and mean SUV of reference region (Aorta). The SUV in different regions were averaged to obtain mean total body SUV values (mean SUV max or mean SUV mean). SUVs in limbs (proximal and distal upper limbs, gluteal, proximal lower limb and calf muscles) were averaged to obtain mean limb SUVs.

SUV mean and SUV max was calculated for all of the above-mentioned muscle groups in the control group. FDG uptake in terms of SUV mean and SUV max was compared between IIM patients and the control group. Considering the physiologically higher FDG uptake in neck muscles in normal healthy people, cervical spinal muscles and sternocleidomastoid muscles were excluded from calculating the mean total body SUVs in the control group. ROC curve was plotted for SUV (for both SUV mean and SUV max separately for total body muscles as well as limb muscles), and optimal SUV cut-off for diagnosing IIM with corresponding sensitivity and specificity was obtained.

The extent of myositis involvement was assessed by visual inspection by nuclear medicine expert (CN) and graded for each of the 7 regions (neck, shoulder, and arm, forearm, thoracic, abdomen, hip and thigh, calf) according to the percentage of length involved (length taken along the long axis of the corresponding region). This myositis extent score was multiplied with SUV mean ratio of that particular region to obtain a composite ‘FDG uptake score’, which gives a quantitative assessment of myositis in PET-MRI. FDG uptake score for all regions were added to obtain ‘Total Body FDG uptake score’, and that for all 4 limbs were added to obtain ‘Limb FDG uptake score’. The FDG uptake scores in limbs and whole body were used as the PET-MRI quantitative measure to assess the influence of various clinical and laboratory parameters. The nuclear medicine expert who analysed PET-MRI images was aware of the clinical diagnosis of IIM, but not about the specific laboratory or histopathological features.

### Muscle MRI and HRCT thorax

MR imaging was performed at a hybrid PET-MR (3 T) imaging facility with simultaneous acquisition capability. The MR imaging protocol sequences included were- Axial T1 weighted, T2 weighted, T2-weighted Fat-saturated. The axial sections were acquired at seven stations from cranio-caudal direction, as determined by coil element spatial sensitivity. The slice thickness was set at 10 mm, the FOV was 330 × 184 mm. The sequence parameters for the T1, T2, T2-fat-saturated axial sections were as follows [TR (ms)/TE (ms)/Voxel dimension (mm)]- 1150 ms/9.7 ms/0.5 × 0.5x10; 4554 ms/99 ms/0.9 × 0.9x10mm; 5010 ms/99 ms/0.9 × 0.9x10mm. Frequency selective fat saturation was applied. The contrast medium administered was Gadopentate dimeglumine (Magnevist, Bayer Yakuhin, Osaka, Japan) or Gadodiamide (Omniscan, Daichii Sankhyo, Tokyo, Japan) at a dose of 0.2 mmol/kg bodyweight.

Muscle MRI with T2-STIR sequence was visually assessed for the presence of muscle hyperintensity suggestive of Myositis. HRCT Thorax was assessed for the presence of features suggestive of Interstitial Lung Disease (ILD).

### Muscle biopsy

Seven patients underwent sampling from quadriceps and three from biceps muscle. The following features were recorded: severity of inflammatory infiltrates, necrosis/regeneration of myofibres, and interstitial edema. The inflammatory infiltrate was categorized into two groups, i.e., sparse or absent (inflammatory cells were absent or present in only few foci) and moderate to severe inflammatory infiltrates (inflammatory cells seen diffusely or in several foci). Similarly, necrosis/regeneration was graded into two categories: absent or minimal myopathic changes, where necrosis or regeneration was completely absent or present only in few scattered areas; and moderate to severe, in which active myopathic changes were seen diffusely or in several fibres. Interstitial edema was categorised into two groups based on its presence or absence.

### Serum autoantibodies

Autoantibodies included MSAs, MAAs, anti-dsDNA and ANA. Anti-dsDNA, MSAs including antibodies against Mi2B, Mi2A, SRP, Jo-1, PL-7, PL-12, TIF-1gamma, NXP-2, EJ, OJ, and MAAs including PM-Scl, Ro-52, SS-A, SS-B, nRNP-Sm, PCNA were identified using Immunoblot technique. ANA was done using the indirect immunofluorescence method.

### Statistical analysis

All the relevant data were analysed using IBM SPSS Statistics Version 26. FDG uptake was compared among patients with different clinical, histological and serological profiles. ROC curve analysis was done to identify the sensitivity and specificity of PET-MRI in diagnosing IIM. Association between FDG uptake and various clinical, laboratory and histopathology parameters were assessed using univariate (unpaired t test and ANOVA) and multivariate (multiple linear regression) analysis. Pearson correlation was used to analyse degree of correlation between FDG uptake and muscle MRI. All analysis with *p* value < 0.05 was considered statistically significant.

## Results

### Patient characteristics

The M:F ratio was 19:11. The age at presentation ranged from 16 to 65 years (mean-40.33); for males it was 16 to 65 years (mean-33.73), whereas for females it was 25 to 55 years (mean-44.16). The mean duration of illness was 7 months (males-6.1; females-8). The clinical and immunological diagnoses were DM = 18; OM = 7; IMNM = 4; PM = 1.

**Fig. 1:**
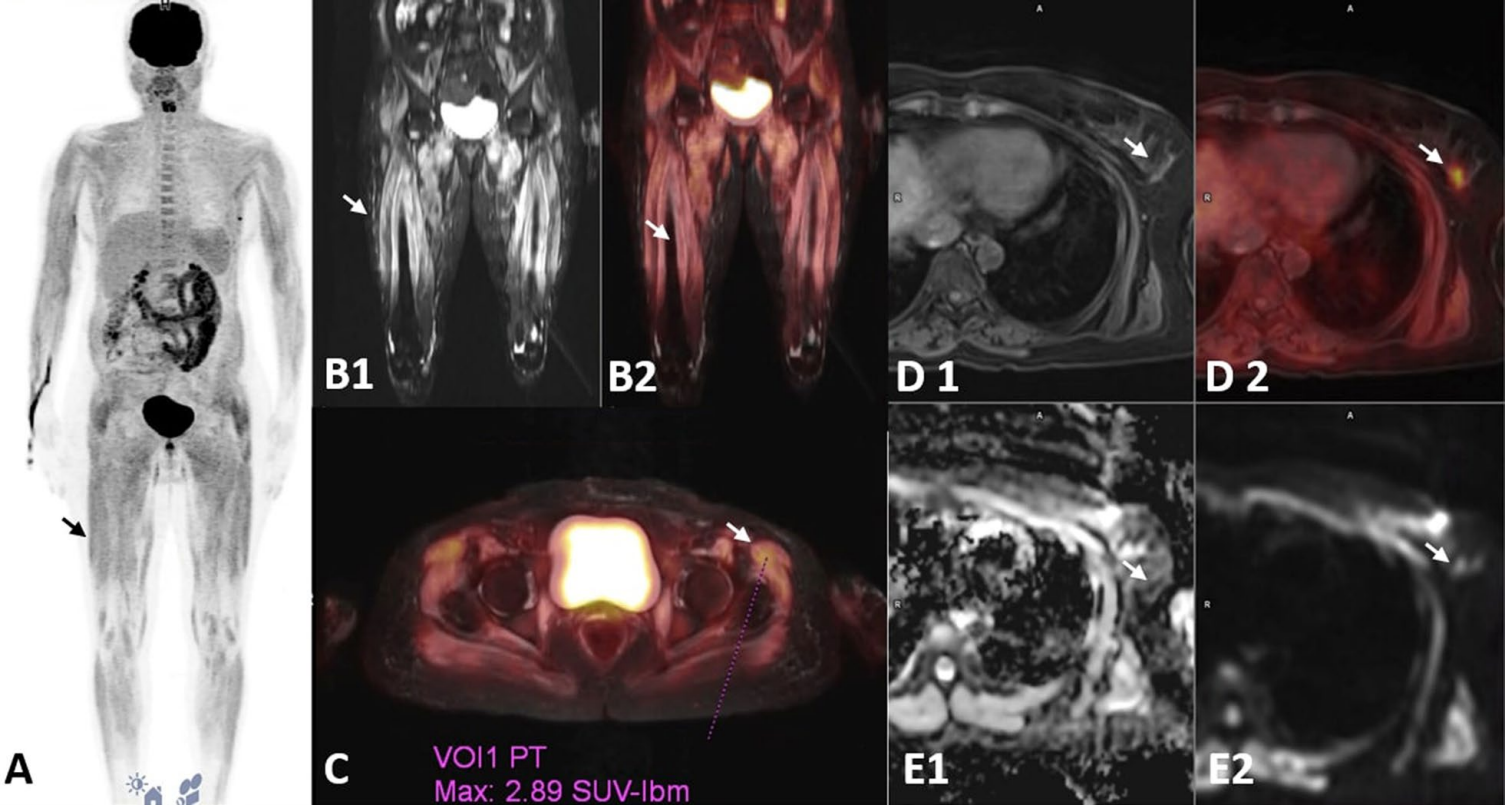
PET-MRI images of a 48-year-old lady with Dermatomyositis and Carcinoma Breast. **A** Whole body MIP image (black arrow). **B1** Coronal T2 FS BLADE MR image showing hyperintensity changes in the proximal thigh muscles (white arrow). **B2** Coronal fused PET/MRI image shows increased FDG uptake in all the proximal thigh muscles (white arrow). **C** Axial Fused T2FS BLADE MR/PET images showing increased FDG uptake in the hip girdle muscles with SUV max of 2.89 (white arrow). **D1** and **D2**. Axial Fused T1 VIBE DIXON (W) and fused PET/MR images showing focal lesion with increased FDG uptake in lower outer quadrant of the left breast (white arrow). **E1** and **E2**. Axial ADC and TRACE DWI MR images showing focal area of diffusion restriction corresponding to the focal lesion on PET (white arrow)

**Fig. 2:**
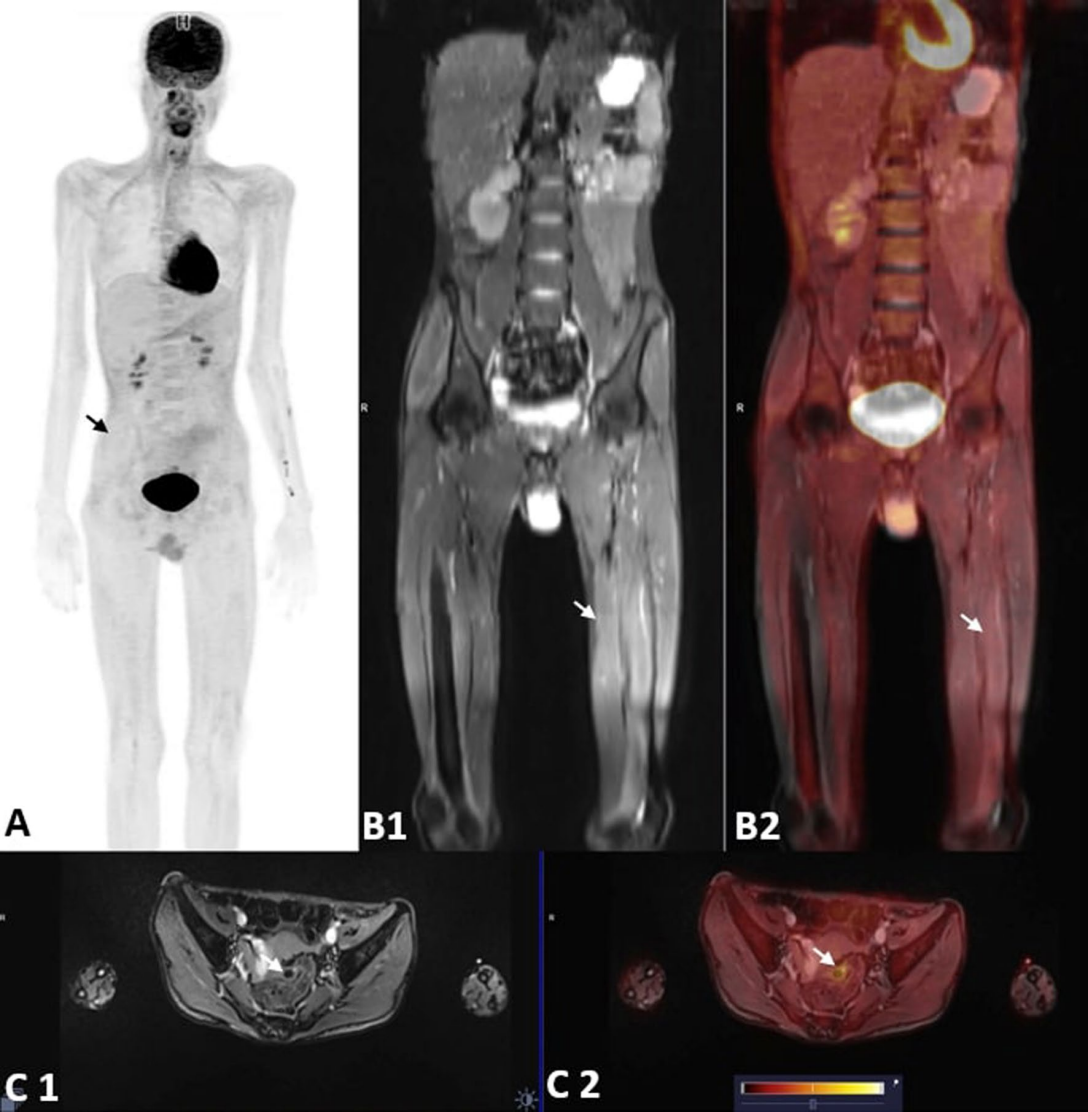
PET-MRI images of a 16-year-old boy with Overlap Myositis. **A** Whole body MIP image (black arrow). **B1** Coronal T2 FS BLADE MR image showing hyperintensity changes in the muscles around hip and lower limbs (white arrow). **B2** Coronal fused PET/MRI image shows increased FDG uptake in all the muscles at HIP and lower limbs (white arrow). **C1** and **C2**. Axial T2FS BLADE MR showing subtle hyperintensity changes in the gluteal muscles (white arrow). Axial Fused PET/MR images showing mild diffuse increased FDG uptake in gluteal muscles (white arrow)

Among the various IIM subcategories, DM patients had the highest seropositivity for MSAs (15/18, 83.33%), whereas maximum MAA positivity was seen in OM (5/7, 83.3%). Most common MSA detected was Mi2B (13/30, 43.33%) and the most common MAA detected was Ro-52 (12/30, 40%). Patients seropositive for ANA had a relatively younger age of disease onset (34.5 ± 11.86 years), greater female predilection (F:M = 3:1), and associated joint pains and photosensitivity (Figs. 1, 2).

**Fig. 3:**
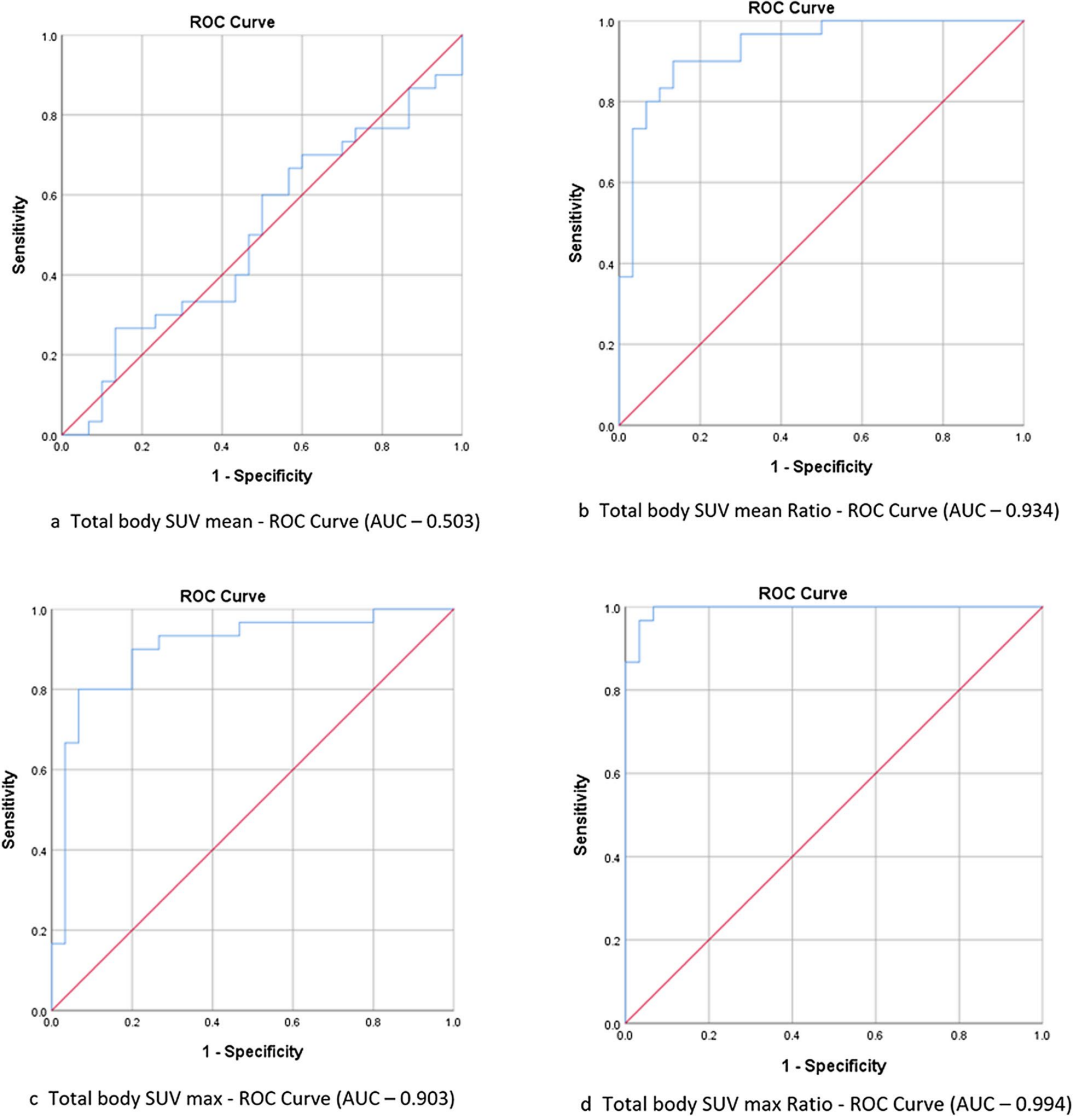

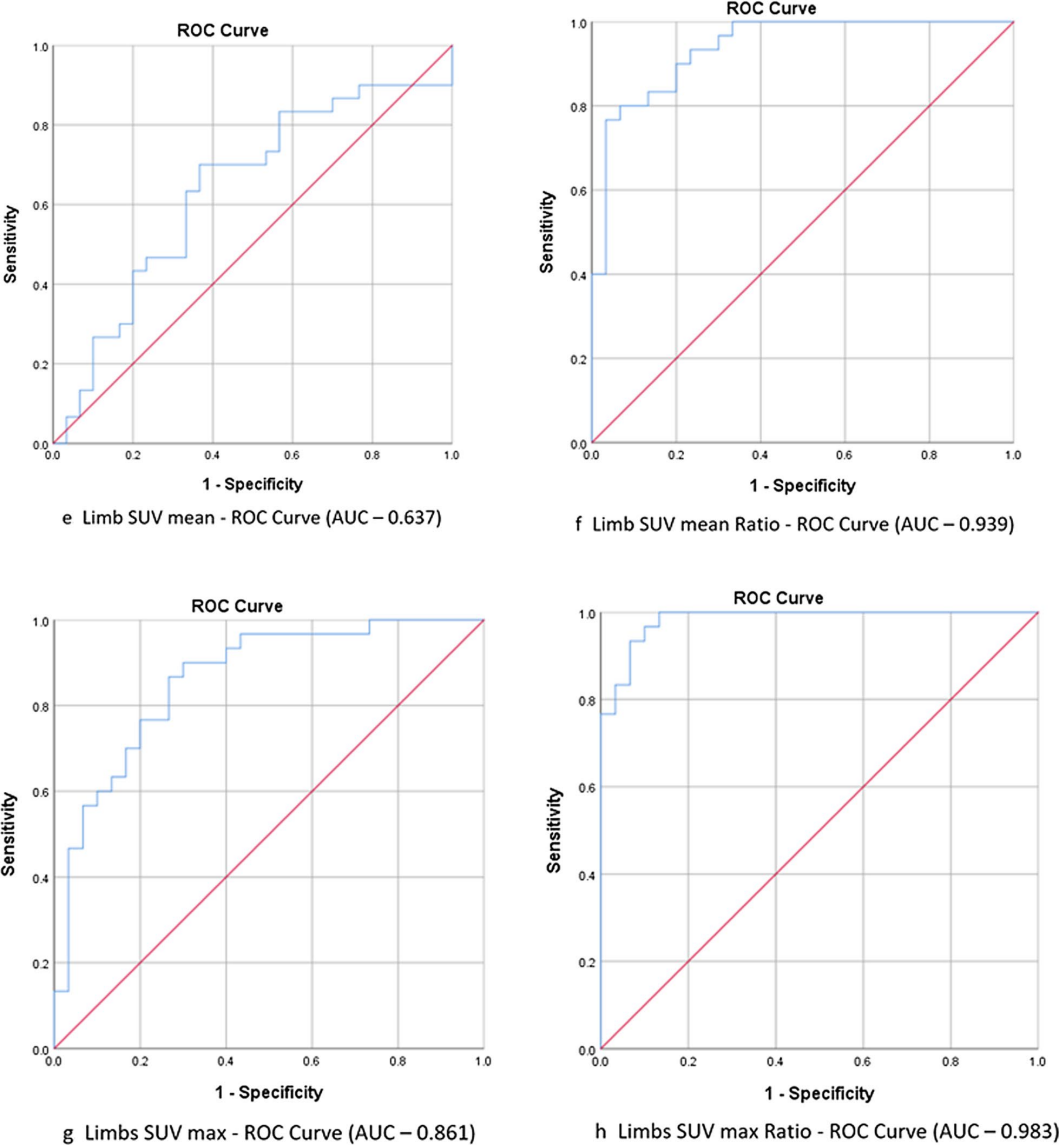
**a**–**d** ROC Curves for FDG uptake in limb muscles using various parameters. **e**–**h** ROC Curves for FDG uptake in total body muscles using various parameters

The mean serum CK (6532 v/s 2278 Units/Litre) and SGOT (232 v/s 87 Units/Litre) values were highest in DM and lowest in OM. Among the various clinical features, facial weakness and respiratory muscle involvement were more prevalent in DM. The salient demographic, clinical and laboratory features are summarised in Table 1 and Additional file 3: Table 1. No adverse effects related to PET-MRI imaging was noted in our study group (Fig. 3).

**Table 1:** Patient characteristics for subcategories of inflammatory myositis

Clinical attribute	Total patients (n = 30)	Dermatomyositis (n = 18) (60%)	Polymyositis (n = 1) (3.33%)	Immune mediated necrotizing myositis (n = 4) (13.33%)	Overlap myositis (n = 7) (23.33%)
Female: male ratio	1.73:1	1.25:1	1:0	3:1	2.5:1
Mean age (years)	40.33 ± 11.56	38.61 ± 10.01	45	44.75 ± 8.54	41.57 ± 17.27
Mean duration of illness (months)	7.3 ± 6.02	7.06 ± 6.40	12 ± 0	5.75 ± 2.5	8.14 ± 7.0
MSA positive	21 (70.00)	15 (83.33) Mi2b = 13 Mi2a = 4 PL7 = 1 PL12 = 1 Tif1gamma = 1 NxP2 = 1		4 (100.0) SRP = 4	2 (28.57) PL7 = 2 PL12 = 1
MAA positive	11 (36.67)	3 (16.67) Ro52 = 4 PM-Scl = 1 PCNA = 1 SS-A = 1 SS-B = 1	1 Ro52 = 1	2 (50) Ro52 = 2	5 (71.43) Ro52 = 5 PM-Scl75 = 1 PM-Scl100 = 1 SS-A = 1 SS-B = 1
ANA positive	8 (26.67)	3 (16.67)	–	1(25.00)	4 (57.14)
Mean serum CK (units/litre ± SD)	5398 ± 4677	6535 ± 5305	3857 ± 0	6127 ± 2859	2278 ± 2435
Mean SGOT (units/litre ± SD)	187 ± 189	232 ± 231	158 ± 0	171 ± 36	87 ± 52
Mean SGPT (units/litre ± SD)	116 ± 80	128 ± 90	148 ± 0	145 ± 41	66 ± 57
Muscle pain	25 (83.33)	16 (88.88)	1	3 (75.00)	5 (71.43)
Muscle cramps	1 (3.33)	1 (5.56)	–	–	–
Limb weakness	30 (100.00)	18 (100.00)	1	2 (100.0)	6 (100.0)
Proximal UL weakness	20 (66.67)	11 (61.11)	1	2 (50.00)	6 (85.71)
Distal UL weakness	7 (23.33)	4 (22.22)	–	–	3 (42.86)
Proximal LL weakness	20 (66.67)	12 (66.67)	1	2 (50.00)	5 (71.43)
Distal LL weakness	4 (13.33)	3 (16.67)	–	–	1 (14.29)
Truncal weakness	19 (63.33)	12 (66.67)	–	3 (75.00)	4 (57.14)
Neck flexor weakness	22 (73.33)	16 (88.89)	–	2 (50.00)	4 (57.14)
Neck extensor weakness	19 (63.33)	14 (77.78)	–	2 (50.00)	3 (42.86)
Bulbar weakness	15 (50.00)	10 (55.56)	1	1	3 (42.86)
Facial weakness	8 (26.67)	8 (44.44)	–	–	–
Respiratory symptoms	3 (10.00)	3 (16.67)	–	–	–
Skin involvement	23 (76.67)	16 (88.89)	0	2 (50.00)	5 (71.43)
Skin Hyperpigmentation	19 (63.33)	13 (72.22)	–	1 (25.00)	5 (71.43)
Photosensitivity	5 (16.67)	3 (16.67)	–	–	2 (28.57)
Scleroderma	7 (23.33)	5 (27.78)	–	–	2 (28.57)
Skin ulcers	1 (3.33)	–	–	–	1 (14.29)
Oral ulcers	1 (3.33)	1 (5.56)	–	–	–
Arthralgia	11 (36.67)	8 (44.44)	–	1	2 (28.57)
Weight loss	15 (50.00)	9 (50.00)	1	2 (50.00)	3 (42.86)
Loss of appetite	10 (33.33)	7 (38.89)	1	1 (25.00)	1 (14.29)
Muscle wasting	15 (50.00)	10 (55.56)	1	1 (25.00)	3 (42.86)
Wheel chair bound at evaluation	14 (46.67)	9 (50.00)	1	2 (50.00)	2 (28.57)

Wherever applicable percentages are mentioned in parentheses

*MSA* Myositis Specific Antibody, *MAA* Myositis Associated Antibody, *ANA* Anti Nuclear Antibody, *CK* Creatine Kinase, *SGOT* Serum Glutamic Oxaloacetate Transaminase, *SGPT* Serum Glutamic Pyruvic Transaminase

### FDG uptake in skeletal muscles

**Fig. 4:**
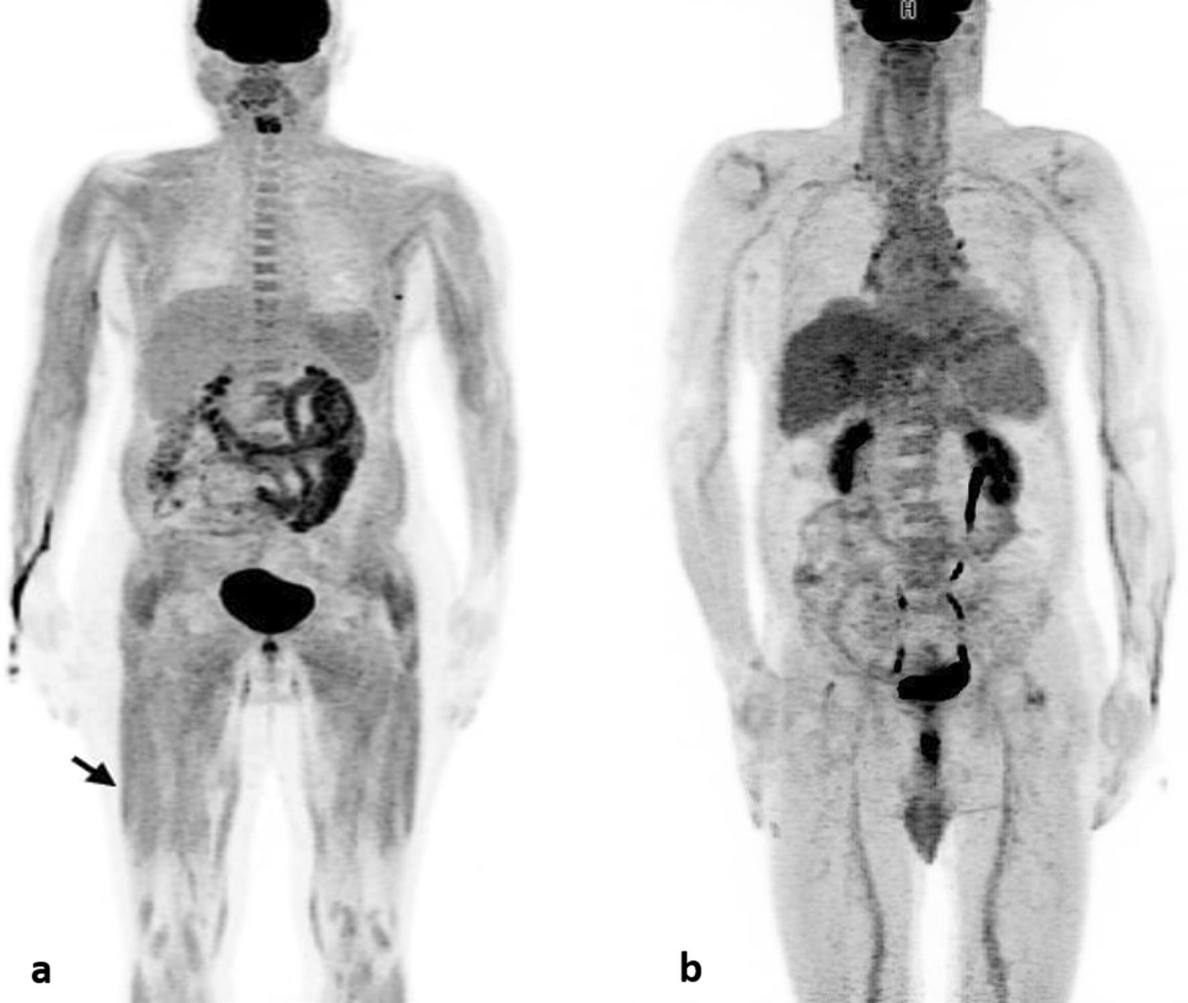
MIP images of patient with Inflammatory Myositis (left panel) and that of a control patient with Amyotrophic Lateral Sclerosis (right panel)

**Fig. 5:**
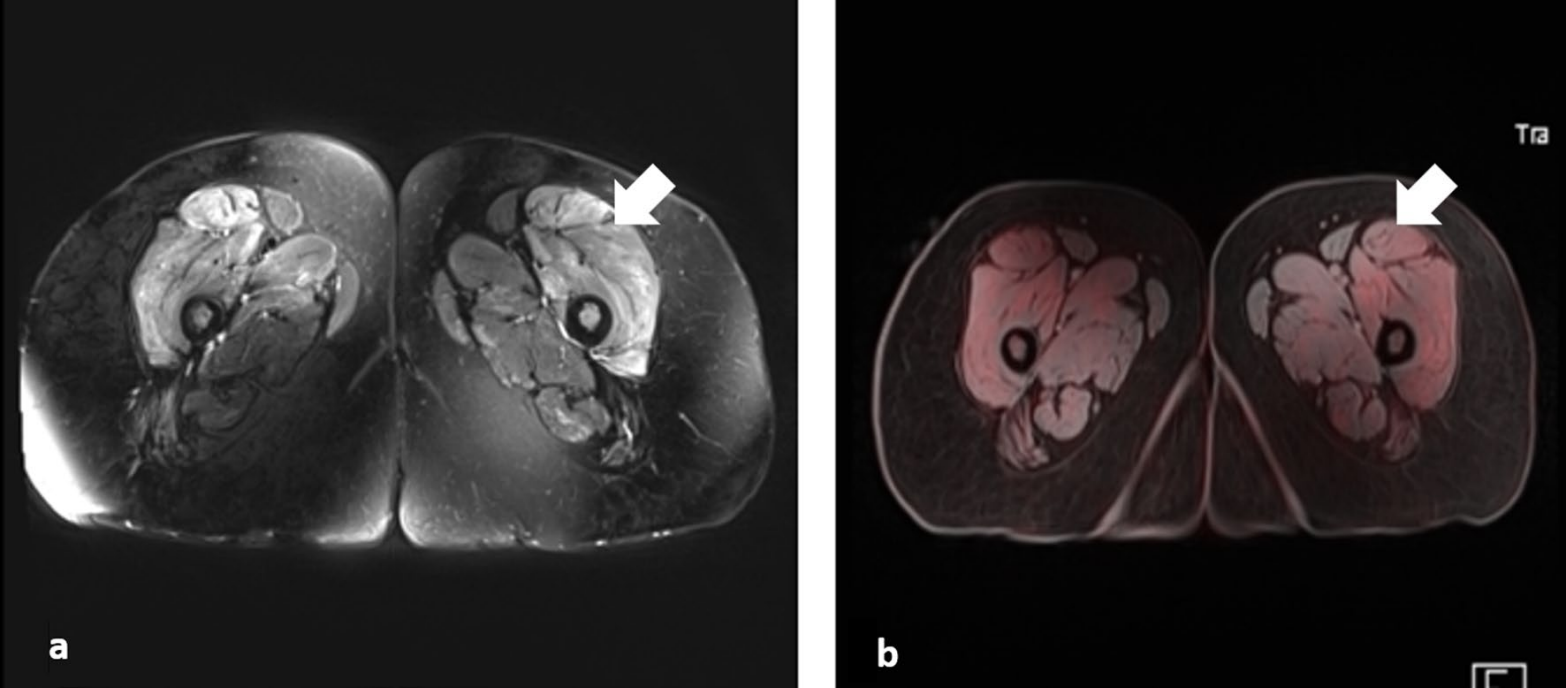
Axial section of thigh muscle in a 33 year old patient with Dermatomyositis showing increased FDG uptake in anterior compartment thigh muscles with corresponding hyperintensity in T2 Fat Saturation sequence.

Myositis was analysed using PET-MRI in 10 regions. The mean FDG uptake for skeletal muscles in IIM patients and controls, combining all of the above regions (Total body SUV) and for combined 4 limbs (Limbs SUV) is given in Table 2. All parameters except total body SUV mean and limbs SUV mean showed a statistically significant increase in FDG uptake in IIM patients. The highest FDG uptake was seen in cervical spinal muscle group (SUV max = 2.39, SUV max Ratio = 2.89), followed by proximal lower limb region (SUV max = 2.05, SUV max ratio = 2.48), whereas paraspinal muscles (SUV max = 1.53, SUV max Ratio = 1.84) showed least mean uptake values (Figs. 4, 5, Additional files 1 and 2 Figs. 1, 2).

**Table 2:** FDG uptake in IIM patients and control group (comparison using unpaired t test)

FDG uptake	Patients	Controls	*p* value
SUV max	1.86 ± 0.38	1.28 ± 0.28	0.000
SUV max ratio	2.30 ± 0.52	0.86 ± 0.26	0.000
SUV mean	0.76 ± 0.15	0.77 ± 0.15	0.866
SUV mean ratio	0.92 ± 0.24	0.52 ± 0.15	0.000
Limbs SUV max	1.79 ± 0.46	1.23 ± 0.33	0.000
Limbs SUV max ratio	2.16 ± 0.66	0.82 ± 0.28	0.000
Limbs SUV mean	0.83 ± 0.18	0.76 ± 0.18	0.111
Limbs SUV mean ratio	1.01 ± 0.29	0.51 ± 0.16	0.000

*p* value < 0.05 considered statistically significant

### Sensitivity and specificity of PET MRI in diagnosing IIM

For calculating the sensitivity and specificity of PET-MRI in diagnosing IIM, ROC curve was plotted for SUV (absolute values and ratio with reference region) in limbs and total muscle groups (Fig. 3). PET-MRI shows a wide range of sensitivity and specificity depending on the parameter used to measure FDG uptake (Additional file 4: Table 2). Overall, SUV max had better sensitivity and specificity compared to SUV mean and, SUV ratios had better sensitivity and specificity compared to absolute SUVs.

### Effect of clinical and laboratory parameters in FDG uptake

The influence of various clinical and laboratory parameters in FDG uptake in PET-MRI was analysed. These included age, sex, duration of illness, clinical diagnosis, severity of muscle weakness, laboratory parameters like ESR, creatinine kinase, transaminases (Serum Glutamic Oxaloacetic Transaminase and Serum Glutamic Pyruvic Transaminase), myositis profile and other autoantibodies. FDG uptake score was used as the quantitative measure for the severity of myositis in PET-MRI. The effect of these variables on FDG uptake is summarised in Table 3. Among the various clinical and laboratory variables, univariate regression analysis showed that clinical diagnosis, severity of limb weakness, presence of MSA, Mi2B positivity, serum CK, SGOT and SGPT had statistically significant association with FDG uptake. When multiple linear regression analysis was done using the variables of clinical diagnosis, severity of muscle weakness, Mi2B positivity and serum CK levels, the latter three were the statistically significant parameters (*p* value of 0.005, 0.043 and 0.042 respectively for total body FDG uptake and 0.002, 0.038 and 0.066 respectively for limb FDG uptake) (Additional file 5: Table 3).

**Table 3:** Clinical/laboratory parameters and FDG uptake score—Univariate Analysis (using unpaired t test and ANOVA)

Clinical parameters	Limbs FDG uptake score	*p* value	Total body FDG uptake score	*p* value
**Age**				
Less than 30 years	10.44 ± 4.17	0.977	17.68 ± 8.31	0.828
30 to 50 years	10.19 ± 2.69		16.3 ± 4.41	
More than 50 years	10.49 ± 4.43		15.85 ± 6.31	
**Duration of illness**				
Less than 6 months	11.16 ± 3.83	0.33	17.97 ± 6.62	0.33
Between 6 to 12 months	9.63 ± 2.13		15.25 ± 3.88	
More than 12 months	8.98 ± 3.27		14.29 ± 4.88	
**Sex**				
Male vs. Female	10.33 ± 3.02 vs. 10.31 ± 3.65	0.99	17.60 ± 6.07 vs. 15.88 ± 5.62	0.44
**Clinical diagnosis**				
Dermatomyositis	11.66 ± 3.34	0.041	19.34 ± 5.2	0.003
Polymyositis	8.89 ± 0		13.5 ± 0	
Necrotizing myositis	9.31 ± 0.42		14.19 ± 1.79	
Overlap myositis	7.64 ± 2.94		10.99 ± 4.11	
**Severity of limb weakness**				
Mild/moderate weakness	9.11 ± 3.06	0.004	14.69 ± 5.27	0.012
Severe weakness	12.74 ± 2.71		20.14 ± 5.09	

Laboratory parameter	Limbs FDG uptake score ± SD	*p* value	Total body FDG uptake score ± SD	*p* value
**Serum autoantibodies**				
Mi2B positivity vs. negative	12.47 ± 3.17 vs. 8.67 ± 2.56	0.001	20.56 ± 5.19 vs. 13.41 ± 4.02	0.000
MSA positive vs. negative	11.21 ± 2.96 vs. 8.24 ± 3.49	0.025	18.16 ± 5.24 vs. 12.68 ± 5.25	0.014
MAA positive vs. negative	7.48 ± 2.81 vs. 11.35 ± 2.99	0.004	11.47 ± 4.08 vs. 18.34 ± 5.19	0.002
ANA positive vs. negative	8.94 ± 3.29 vs. 10.82 ± 3.35	0.183	14.93 ± 6.67 vs. 17.09 ± 5.43	0.372
**ESR**				
Less than 30 mm/hr	10.89 ± 2.84	0.536	17.33 ± 4.78	0.595
More than 30 mm/hr	9.97 ± 4.04		16.01 ± 6.76	
**Creatine Kinase**				
Less than 5 X ULN vs. More than 5 X ULN	6.46 ± 3.77 vs. 10.91 ± 2.97	0.012	9.27 ± 3.92 vs. 17.63 ± 5.18	0.005
**SGOT**				
Normal	7.40 ± 4.05	0.077	10.91 ± 4.59	0.03
Above normal but less than 5 X ULN	10.36 ± 3.07		16.55 ± 5.03	
More than 5 X ULN	12.48 ± 3.00		20.85 ± 6.50	
**SGPT**				
Normal	7.20 ± 3.16	0.007	10.74 ± 3.63	0.002
Above normal but less than 5 X ULN	10.53 ± 2.95		16.84 ± 5.28	
More than 5 X ULN	13.25 ± 2.41		22.18 ± 2.64	

### FDG uptake and histopathological features

The FDG uptake when compared with major pathological characteristics, two variables (degree of inflammation, interstitial edema) showed significant statistical association in terms of regional limb and total body FDG uptake. Other pathological features like vascular changes, myopathic features, necrosis and regeneration did not show any statistically significant correlation with FDG uptake (Additional file 6: Table 4).

### Correlation between muscle MRI and FDG uptake in PET MRI

Thirteen patients underwent muscle MRI with T2-STIR sequence, 11 patients (DM = 9, IMNM = 1, OM = 1) showed STIR hyperintensity corresponding to FDG uptake in PET-MRI. In one patient (DM) with positive FDG uptake, STIR hyperintensity was absent. In another patient (DM), STIR hyperintensity was present in certain muscles matching the increased FDG uptake, but not in other muscles like anterior compartment of thigh.

Regional FDG uptake score calculated in proximal LLs and calf region showed statistically significant association with muscle MRI score in corresponding region (r = 0.579 and *p* value = 0.015 for proximal LLs; r = 0.602 and *p* value = 0.011 for calf region).

### ILD in inflammatory myositis

Twelve patients underwent HRCT chest, of which 4 (25%) had evidence of ILD. They had duration of illness ranging between 4 to 20 months. Two of them were positive for Mi2B, one for PL-7 and one was ANA positive. Three of them had diffuse FDG uptake in lungs in PET MRI. In PET MRI, 7 patients showed increased lung FDG uptake; 4 had diffuse and 3 had focal increase in uptake. There was no difference in muscle FDG uptake between IIM patients with and without ILD.

### Malignancy in inflammatory myositis

Among the 30 patients, three were detected to have primary malignancy. A 48-year-old lady with Mi2B seropositivity, had carcinoma breast on PET-MRI. The other, a 50-year-old lady with OM (Ro52 positive) had uterine carcinoma. The third patient, with SRP positive IMNM had carcinoma cervix diagnosed 2 years before presentation of myositis. FDG uptake in these 3 patients was comparable to that of the remaining patients.

### Treatment response and FDG uptake

Among the patient group, data regarding immediate and long-term treatment was available for 20 patients and long term follow up details were available for 15 patients. In the acute phase of disease, 17 patients (85%) received iv methylprednisolone and 3 15%) received iv methylprednisolone and cyclophosphamide. Among the iv-methylprednisolone group, 3 patients (17.64%) showed good symptomatic response, 8 (47.06%) had no improvement or progressive worsening and 6 (35.29%) showed partial improvement in the immediate post-treatment period. Among the 3 patients in the second group, one had good response, one had partial improvement and the third did not show any response. For long term immunosuppression, 18 patients (90%) received intravenous or oral steroids along with another immunosuppressant, whereas 2 patients (10%) received Rituximab injection. In the long term follow-up group (n = 15), all who received iv cyclophosphamide (n = 9) along with steroids had moderate to good response (9/9, 100%). Among 3 patients who received MMF, 2 had good response whereas one patient showed no improvement. One patient received Hydroxychloroquine and showed no improvement, whereas 2 patients who received oral Methotrexate, were lost for follow-up. Among the patients with initial poor response to iv methylprednisolone (n = 9), long term follow up data was available for 6 patients and 4 of them had good response to long term immunosuppressants (2 received cyclophosphamide and 2 rituximab), whereas 2 patients had no improvement (one was being continued on iv methylprednisolone and other was given MMF). Three patients (3/15, 20%) developed relapse between 5 to 7 months after initial treatment, among whom long term immunosuppressant used was iv cyclophosphamide in 2 patients and oral Hydroxychloroquine in the third.

There was no statistically significant difference in FDG uptake among patients with varying immediate and long-term treatment response. Clinical and laboratory variables including age, sex, duration of illness, severity of muscle weakness, myositis profile, ESR, serum CPK, SGOT and SGPT did not show any significant correlation with treatment response.

PET-MRI was taken before administration of steroid in 13 patients and in the remaining 9 it was done 1 to 5 days after completion of steroid pulse therapy. There was no significant difference in FDG uptake among patients who underwent PET-MRI before and after steroids.

## Discussion

In this study, we evaluated the utility of FDG-PET-MRI in the diagnosis of inflammatory myositis in thirty cases and thirty controls and also aimed to detect underlying malignancy. The sensitivity and specificity of PET-MRI depends on the parameter used to quantify FDG uptake. The total muscle FDG uptake was significantly higher in myositis patients compared to controls, when it was expressed as SUV max, SUV mean Ratio and SUV max Ratio but not when expressed as SUV mean. This can be logically explained by the fact that IIM affects certain muscle groups only, thus nullifying the effect of SUV mean (which is lesser than SUV max for corresponding ROI) when mean value is taken including all the muscle groups in the body. When SUV mean of limbs is calculated, it is significantly higher compared to control group. Another important finding was that the difference in FDG uptake was more evident when expressed in SUV Ratio rather than absolute SUVs. This likely reflects the difference in basal body metabolic rate between patients and control group, which is corrected by obtaining the ratio with reference region. Most of the previous studies have shown a higher FDG uptake using PET-CT compared to controls [13–16].

Sensitivity and specificity of PET-MRI in diagnosing IIM depends on the parameter used to quantify FDG uptake. The sensitivity ranges from 60 to 100% and specificity from 50 to 100%. We observed that when SUV max Ratio cut-off was taken at 1.259, sensitivity and specificity were 100% and 93.3% respectively, whereas when 1.684 was taken as the cut-off, sensitivity and specificity was 86.9% and 100% respectively. Previous studies show different sensitivity and specificity for PET-CT in IIM, which varied based on the methodology adopted for quantifying FDG uptake. In a retrospective study by Tanaka et al., the optimal cut-point was determined by the ROC curve in proximal limb muscle at SUV mean of 0.83, giving a sensitivity of 90% and a specificity of 100% [15]. In a study by Okuda et al., when muscle uptake was greater than that of the liver, it was defined as the positivity criterion, and sensitivity was 33% with 97% specificity. This lower sensitivity was possibly due to the methodology adopted in quantifying muscle uptake [17]. Comparing muscle MRI with PET-MRI, we found moderately strong statistically significant correlation between PET-MRI FDG uptake score and STIR hyperintensity in muscle MRI. Among the 13 patients who underwent muscle MRI, STIR hyperintensity was noted in 11 (84.61%) while FDG uptake was increased in 100% of patients. Muscle MRI abnormalities matched with regions of increased FDG uptake in all 11 patients. This strongly suggests that FDG-PET-MRI has greater sensitivity compared to MRI in detecting active myositis in IIM.

For analysing correlation of myositis activity in PET-MRI with other clinical and laboratory variables, we used ‘FDG uptake score’ as the quantitative measure of muscle inflammation in PET-MRI. When FDG uptake was compared between different subcategories of IIM using univariate linear regression analysis, DM patients showed significantly higher uptake than PM, NM or OM, individually or combined while there was no significant difference in FDG uptake among the latter groups. However, in multivariate regression analysis, diagnostic subgroups did not show any statistically significant association with FDG uptake. Both univariate and multivariate regression analysis showed a statistically significant association between clinical severity of limb weakness and FDG uptake. Patients with more severe weakness showed an increase in FDG uptake. Similar positive correlation between clinical muscle strength and FDG uptake using PET-CT is reported in two different studies [14, 15]. However, in another study authors did not find any correlation between muscle strength and FDG uptake [13].

The effect of seropositivity for MSAs or MAAs on FDG uptake was evaluated. There was a significantly higher FDG uptake among MSA positive as compared to MAA positive group. The patients positive for Mi2B had significantly higher FDG uptake in comparison to other groups. Among dermatomyositis patients, Mi2B positive patients had higher FDG uptake compared to Mi2B negative cases, but this was not statistically significant (*p* = 0.069, for total body FDG uptake score). Other antibodies like SRP, PL-7, PL-12 had no effect on extent of FDG uptake. In univariate regression analysis, the presence of MSA, MAA and Mi2B antibodies were statistically significant, however, in multivariate regression analysis, Mi2B positivity was the only significant factor. Among DM patients, those with Mi2B antibodies had significantly higher FDG uptake compared to those without antibodies. No previous studies have compared the effect of serum autoantibodies on FDG uptake.

Among the various laboratory parameters analyzed, serum CK, SGOT and SGPT levels showed a positive correlation with FDG uptake in univariate regression analysis. In multivariate regression, serum CK level was the only significant variable having a correlation with FDG uptake. Lu Sun et al., and Tateyama et al., showed a positive correlation between FDG uptake and serum CK level, whereas Pipitone et al., did not find any such correlation [13, 14, 16]. Tanaka et al., showed a positive correlation of FDG uptake with serum CK and aldolase levels [15].

The histopathological findings also tend to have a positive correlation with FDG uptake. The severity of muscle inflammatory infiltrates, and interstitial edema had a moderately strong positive correlation with FDG uptake. This indicates that FDG-PET-MRI adequately captures these events in the process of muscle inflammation. The inflammatory infiltrates composed of lymphocytes and macrophages might probably be providing the substrate for the observed increase in FDG uptake. However, we could not find any statistically significant difference in FDG uptake in patients based on the severity of necrosis/regeneration. Tanaka et al., observed a positive correlation between the severity of inflammatory infiltrates and FDG uptake, whereas Tatayama et al., reported a positive correlation between FDG uptake with the severity of inflammatory infiltrates and necrosis/regeneration ^15,16^.

At the time of diagnosis, only three (10%) patients had evidence of primary malignancy on PET-MRI. First patient had DM with Mi2B seropositivity, second with OM for Ro52 antibody and third patient with SRP positive IMNM with carcinoma cervix 2 years before onset of myositis. The FDG uptake in these patients were comparable to the mean uptake of remaining patient group. Among the various subcategories of IIM, DM is reported to have a stronger association with malignancy than PM (32% vs. 15%), with 60% of these cases in DM being diagnosed later during follow up. The prevalence of malignancy in our patient cohort is far less but may increase during subsequent follow up scanning.

Most of the patients received intravenous methylprednisolone and showed either moderate to good response. The poor responders received cyclophosphamide or rituximab as long-term immunosuppressant and showed good clinical response. Intriguingly, no significant correlation between FDG uptake in PET MRI and subsequent treatment response was noted. Similarly, no clinical or laboratory variable showed any significant association with treatment response. The dissociation between treatment response and severity of myositis assessed using FDG uptake score in PET MRI possibly indicates heterogeneity in the basic pathogenesis in the study group. From these observations, we can speculate the disease probably manifests along a spectrum of varying degrees of inflammation and clinical/laboratory parameters, but the underlying disease pathophysiology has no significant correlation with these observed variables. Further studies with a larger sample size could help in confirming this hypothesis.

The limitations of this study include the lower sample size of the patient group. The Control group was not ideal healthy age-matched individuals. A significant proportion of patients underwent PET-MRI after starting treatment with immunosuppressants, which can confound results by modifying the inflammatory response. Pathological biopsy was performed in only 10 patients, and only 13 patients underwent muscle MRI with T2 STIR sequences. Since the study was retrospective, data related to clinical examination was obtained from patient records.

## Conclusion

PET-MRI is a promising diagnostic modality for IIM with good sensitivity and specificity. PET-MRI reflects the severity of muscle inflammation, showing good association with various clinical/laboratory parameters, histopathology and muscle MRI. Parameters associated with severe muscle inflammation in PET-MRI in our study includes—clinical severity of muscle weakness, Mi2B positivity and serum creatine kinase levels. PET-MRI has the added advantage of detection of systemic malignancy in IIM patients.

## Supplementary Information

**Additional file 1: Figure 1**. A 53-year-old lady with Necrotizing Myositis. Legends—A. Whole body MIP image (black arrow). B1. Coronal fused PET/MRI image shows increased FDG uptake in all the muscles of the body (white arrow). B2. Coronal T2 FS BLADE MR image shows hyperintensity in the muscles of the body (white arrow).C1 and C2. Axial fused PET/MRI and T2FS BLADE MR showing increased FDG uptake in the mid lower leg level involving the medial compartment muscles along with subtle hyperintensity changes (white arrow). C3: Coronal fused PET MRI images shows increased tracer uptake in the gastrocnemius muscle with SUV max 1.55 (white arrow). D1, D2, D3: Axial Fused PET/MR images showing increased FDG uptake in mediastinal nodes and chest and shoulder girdle muscles (white arrow).

**Additional file 2: Figure 2**. A 28-year-old man with Dermatomyositis. Legends—A. Whole body MIP image (black arrow). B. Coronal fused PET/MR image showing increased FDG uptake in all the muscles of the body (white arrow). C. Coronal T1 VIBE DIXON (W) MR images showing hyperintensity changes in the muscles (white arrow). D. Axial Fused PET/MR images showing bilateral mildly hypermetabolic mediastinal nodes (white arrow). E. Axial fused PET/MR images at the level of proximal thigh showing increased FDG uptake in muscles of the medial compartment of the thigh with SUV max of 1.56(white arrow). F. Axial fused PET/MR images showing focal increased FDG uptake in the left rib likely benign fracture (white arrow).

**Additional file 3: Table 1**. Characteristic features of Inflammatory Myositis patients based on serum autoantibody profile.

**Additional file 4: Table 2**. Sensitivity and Specificity of PET-MRI (SUV of total muscle groups) at various cut-off points.

**Additional file 5: Table 3**. Multivariate analysis of various clinical and laboratory parameters.

**Additional file 6**. Pathological features showing association with FDG uptake.

## Data Availability

Preliminary clinical, laboratory and imaging data used in this study will be made available for anyone at reasonable request.
