# Supplementary figures and images for: PET-MRI in idiopathic inflammatory myositis: a comparative study of clinical and immunological markers with imaging findings
Source: Neurol Res Pract. 2022 Oct 10;4:49. doi: 10.1186/s42466-022-00213-9 (PMC9549636; doi:10.1186/s42466-022-00213-9)

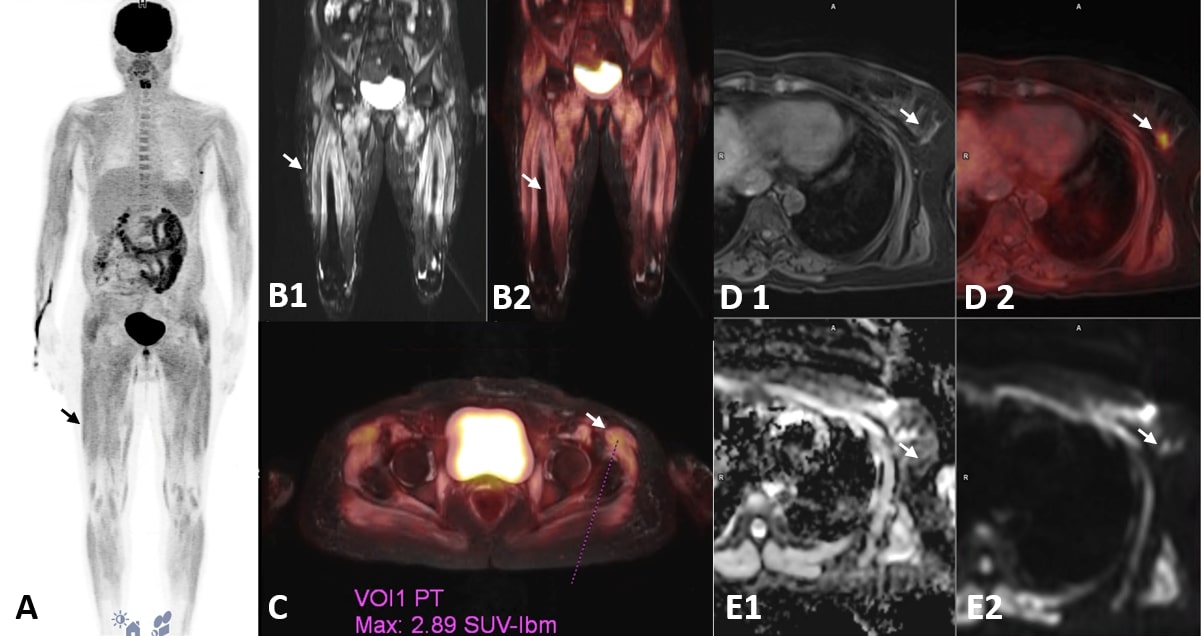

Supplement: Supplementary file 1 — Additional file 1: Figure 1. A 53-year-old lady with Necrotizing Myositis. Legends—A. Whole body MIP image (black arrow). B1. Coronal fused PET/MRI image shows increased FDG uptake in all the muscles of the body (white arrow). B2. Coronal T2 FS BLADE MR image shows hyperintensity in the muscles of the body (white arrow).C1 and C2. Axial fused PET/MRI and T2FS BLADE MR showing increased FDG uptake in the mid lower leg level involving the medial compartment muscles along with subtle hyperintensity changes (white arrow). C3: Coronal fused PET MRI images shows increased tracer uptake in the gastrocnemius muscle with SUV max 1.55 (white arrow). D1, D2, D3: Axial Fused PET/MR images showing increased FDG uptake in mediastinal nodes and chest and shoulder girdle muscles (white arrow). [file 42466_2022_213_MOESM1_ESM.jpg]

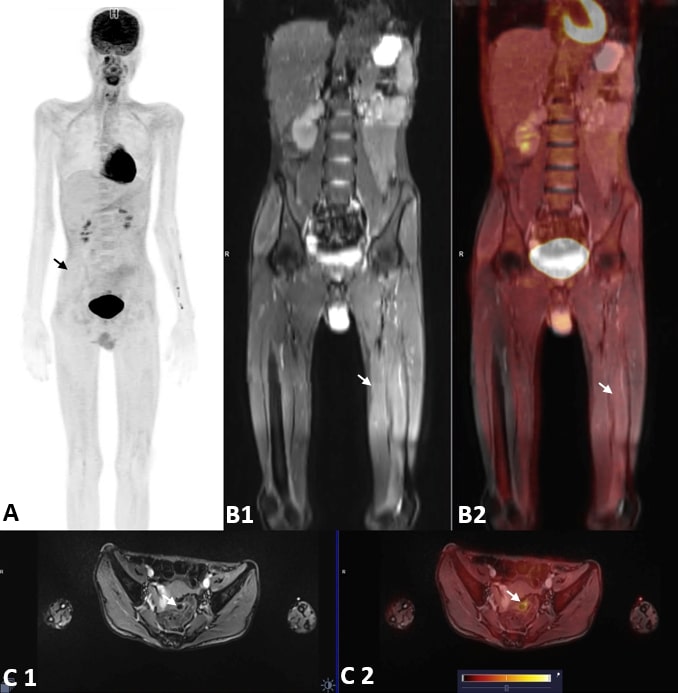

Supplement: Supplementary file 2 — Additional file 2: Figure 2. A 28-year-old man with Dermatomyositis. Legends—A. Whole body MIP image (black arrow). B. Coronal fused PET/MR image showing increased FDG uptake in all the muscles of the body (white arrow). C. Coronal T1 VIBE DIXON (W) MR images showing hyperintensity changes in the muscles (white arrow). D. Axial Fused PET/MR images showing bilateral mildly hypermetabolic mediastinal nodes (white arrow). E. Axial fused PET/MR images at the level of proximal thigh showing increased FDG uptake in muscles of the medial compartment of the thigh with SUV max of 1.56(white arrow). F. Axial fused PET/MR images showing focal increased FDG uptake in the left rib likely benign fracture (white arrow). [file 42466_2022_213_MOESM2_ESM.jpg]
