# Supplementary material for: PET-MRI in idiopathic inflammatory myositis: a comparative study of clinical and immunological markers with imaging findings
Source: Neurol Res Pract. 2022 Oct 10;4:49. doi: 10.1186/s42466-022-00213-9 (PMC9549636; doi:10.1186/s42466-022-00213-9)
Supplement: Supplementary file 3 — Additional file 3: Table 1. Characteristic features of Inflammatory Myositis patients based on serum autoantibody profile. [file 42466_2022_213_MOESM3_ESM.docx]

**Supplementary Table 1- Characteristic features of Inflammatory Myositis patients based on serum autoantibody profile.**

| **Clinical Attribute** | **Total Patients (n = 30)** | **MSA Positive (n = 21)** | **MAA Positive (n = 9)** | **ANA Positive (n = 8)** |
| --- | --- | --- | --- | --- |
| F:M | 1.73:1 | 1.63:1 | 2:1 | 3:1 |
| Mean age (in years, ±SD) | 40.33 ± 11.56 | 39.43 ± 10.04 | 42.44 ± 15.01 | 34.5 ± 11.86 |
| Mean duration of illness (in months, ±SD) | 7.3 ± 6.02 | 5.67 ± 5.19 | 11.11 ± 6.35 | 7.62 ± 7.27 |
| Mean Serum CK (±SD) | 5398 ± 4677 | 6867 ± 4737 | 1970 ± 2079 | 4118 ± 4075 |
| Mean SGOT (±SD) | 187 ± 189 | 235 ± 207 | 76 ± 52 | 149 ± 103 |
| Mean SGPT (±SD) | 116 ± 80 | 14 ± 78 | 56 ± 48 | 114 ± 117 |
| Muscle pain | 25 (83.33) | 18 (85.71) | 7 (77.78) | 5 (83.3) |
| Muscle Cramps | 1 (3.33) | 1 (4.76) | - | - |
| Muscle tenderness | 9 (30.00) | 7 (33.33) | 2 (22.22) | 3 (37.25) |
| Limb weakness | 30 (100.00) | 21 (100.00) | 9 (100.0) | 8 (100.0) |
| Proximal UL weakness | 20 (66.67) | 19 (90.48) | 9 (100.0) | 8 (100.0) |
| Distal UL weakness | 7 (23.33) | 11 (52.38) | 4 (44.44) | 2 (25.00) |
| Proximal LL weakness | 20 (66.67) | 20 (95.23) | 8 (88.89) | 7 (87.50) |
| Distal LL weakness | 4 (13.33) | 8 (38.09) | 1 (11.11) | 2 (25.00) |
| Neck flexor weakness | 22 (73.33) | 16 (76.19) | 6 (66.67) | 6 (75.00) |
| Neck extensor weakness | 19 (63.33) | 13 (53.8) | 6 (66.67) | 4 (50.00) |
| Truncal weakness | 19 (63.33) | 14 (66.67) | 5 (55.55) | 5 (62.50) |
| Facial weakness | 8 (26.67) | 6 (28.57) | 2 (22.22) | 2 (25.00) |
| Bulbar weakness | 15 (50.00) | 9 (42.86) | 6 (66.67) | 4 (50.00) |
| Respiratory symptoms | 3 (10.00) | 2 (9.52) | 1 (11.11) | 1 (12.50) |
| Skin involvement | 23 (76.67) | 18 (85.71) | 5 (55.56) | 7 (87.50) |
| Hyperpigmentation | 19 (63.33) | 14 (66.67) | 5 (55.56) | 6 (75.00) |
| Scleroderma | 7 (23.33) | 4 (19.05) | 3 (33.33) | 2 (25.00) |
| Skin ulcers | 1 (3.33) | - | 1 (11.11) | - |
| Photosensitivity | 5 (16.67) | 2 (9.52) | 3 (33.33) | 3 (37.50) |
| Arthralgia | 11 (36.67) | 7 (33.33) | 4 (44.44) | 5 (62.50) |
| Oral ulcers | 1 (3.33) | - | 1 (11.11) | 1 (12.50) |
| Weight loss (>10% within last 6 months) | 15 (50.00) | 9 (42.86) | 6 (66.67) | 4 (50.00) |
| Loss of appetite | 10 (30.00) | 6 (28.57) | 4 (44.44) | 3 (37.25) |
| Muscle wasting | 15 (50.00) | 8 (44.44) | 7 (77.78) | 3 (37.50) |
| Wheel chair bound | 14 (46.67) | 10 (47.61) | 4 (44.44) | - |

*MSA – Myositis Specific Antibodies; MAA – Myositis Associated Antibodies; ANA – Anti Nuclear Antibody.*

*Patients with both MSA and MAA were included in MSA positive group; MAA positive group is comprised of patients with only MAA.*
