# Supplementary material for: PET-MRI in idiopathic inflammatory myositis: a comparative study of clinical and immunological markers with imaging findings
Source: Neurol Res Pract. 2022 Oct 10;4:49. doi: 10.1186/s42466-022-00213-9 (PMC9549636; doi:10.1186/s42466-022-00213-9)
Supplement: Supplementary file 4 — Additional file 4: Table 2. Sensitivity and Specificity of PET-MRI (SUV of total muscle groups) at various cut-off points. [file 42466_2022_213_MOESM4_ESM.docx]

**Supplementary Table 2 - Sensitivity and Specificity of PET-MRI (SUV of total muscle groups) at various cut-off points.**

| **Cut-off Value** | **Sensitivity (%)** | **Specificity (%)** | **Area Under Curve** |
| --- | --- | --- | --- |
| Total body SUV mean – 0.737 | 60.00 | 50.00 | 0.503 |
| Total body SUV mean Ratio – 0.67 | 90.00 | 88.77 | 0.934 |
| Total body SUV max – 1.583 | 80.00 | 93.33 | 0.903 |
| Total body SUV max Ratio – 1.684 | 86.90 | 100.00 | 0.994 |
| Limb SUV mean – 0.765 | 70.00 | 63.33 | 0.637 |
| Limb SUV mean Ratio – 0.755 | 80.00 | 93.33 | 0.939 |
| Limb SUV max – 1.329 | 90.00 | 70.00 | 0.861 |
| Limb SUV max Ratio – 1.149 | 100.00 | 86.70 | 0.983 |
