# Supplementary material for: PET-MRI in idiopathic inflammatory myositis: a comparative study of clinical and immunological markers with imaging findings
Source: Neurol Res Pract. 2022 Oct 10;4:49. doi: 10.1186/s42466-022-00213-9 (PMC9549636; doi:10.1186/s42466-022-00213-9)
Supplement: Supplementary file 5 — Additional file 5: Table 3. Multivariate analysis of various clinical and laboratory parameters. [file 42466_2022_213_MOESM5_ESM.docx]

**Supplementary Table 3 – Multivariate analysis of various clinical and laboratory parameters.**

| **Variable** | **p value (Limb FDG uptake)** | **p value (Total body FDG uptake)** |
| --- | --- | --- |
| Diagnostic Group | 0.648 | 0.165 |
| Severity of limb weakness | 0.002 | 0.005 |
| Mi2B positivity | 0.038 | 0.043 |
| Serum Creatinine Kinase levels | 0.066 | 0.042 |

*Regression coefficients- For Limb FDG uptake, R = 0.778 and R square = 0.605; For Total body FDG uptake, R = 0.813 and R square = 0.662*
