# Supplementary material for: PET-MRI in idiopathic inflammatory myositis: a comparative study of clinical and immunological markers with imaging findings
Source: Neurol Res Pract. 2022 Oct 10;4:49. doi: 10.1186/s42466-022-00213-9 (PMC9549636; doi:10.1186/s42466-022-00213-9)
Supplement: Supplementary file 6 — Additional file 6. Pathological features showing association with FDG uptake. [file 42466_2022_213_MOESM6_ESM.docx]

**Supplementary Table 4– Pathological features showing association with FDG uptake**

| **Pathological feature** | **Regional FDG uptake** | **p value** | **Limb FDG uptake** | **p value** | **Total Body FDG uptake** | **p value** |
| --- | --- | --- | --- | --- | --- | --- |
| **Degree of inflammation** |  |  |  |  |  |  |
| Absent or mild vs. moderate to severe | 1.76 ± 0.62 vs. 3.50 ± 0.91 | 0.017 | 7.04 ± 3.83 vs.12.54 ± 2.95 | 0.037 | 11.38 ± 6.39 vs. 20.58 ± 3.9 | 0.021 |
| **Interstitial Edema** |  |  |  |  |  |  |
| Absent vs. present | 1.76 ± 0.62 vs. 3.50 ± 0.91 | 0.017 | 7.04 ± 3.83 vs. 12.54 ± 2.95 | 0.037 | 11.38 ± 6.39 vs. 20.58 ± 3.9 | 0.021 |

*P value < 0.05 considered statistically significant*
